# Development of Postoperative Pneumonia After Endovascular Aortic Aneurysm Repair is Associated with an Increased Length of Intensive Care Unit Stay

**DOI:** 10.7759/cureus.4514

**Published:** 2019-04-21

**Authors:** Cam Dung Le, Erik Lehman, Faisal Aziz

**Affiliations:** 1 Surgery, Penn State College of Medicine, Penn State Milton S. Hershey Medical Center, Hershey, USA; 2 Cardiac / Thoracic / Vascular Surgery, Penn State College of Medicine, Penn State Milton S. Hershey Medical Center, Hershey, USA

**Keywords:** endovascular aneurysm repair (evar), pneumonia, abdominal aortic aneurysm (aaa), icu length of stay

## Abstract

Objective

Endovascular aortic aneurysm repair (EVAR) has increasingly replaced open aortic surgery for treatment of abdominal aortic aneurysms (AAA). One of the key advantages of EVAR is the reduced length of intensive care unit (ICU) stay and hospital stay. This study aimed to identify the risk factors associated with increased ICU length of stay (LOS) after EVAR.

Methods

The American College of Surgeons (ACS-NSQIP) database for the year 2013 was used. All patients who underwent EVAR were divided into two groups: ICU LOS <1 day vs. ≥1 day. Preoperative, intraoperative, and postoperative factors were compared between these two groups utilizing bivariate logistic regression analysis. Multivariable logistic regression analysis was then used to identify factors that were independently associated with ICU LOS ≥1 day after EVAR.

Results

A total of 2,468 patients (18.7% females, 81.3% males) were identified. Group 1 (ICU LOS <1 day) = 1,535 patients and Group 2 (ICU LOS ≥1 day) = 933 patients. Multivariable analysis identified the following factors to be associated with ICU LOS ≥1 day: ruptured AAA (OR 3.88, CI 1.97-7.65), the American Society of Anesthesiology (ASA) score of 4-5 (OR 2.82, CI 1.50-5.31), operative time ≥180 minutes (OR 2.10, CI 1.51-2.93), bilateral groin cut down (OR 1.37, CI 1.10-1.71), juxta-renal AAA (OR 1.65, CI 1.16-2.35), renal artery stent (OR 2.13, CI 1.42-3.21), aortic stent (OR 2.39, CI 1.60-3.55), emergency surgery (OR 2.56, CI 1.94-3.38), need for blood transfusion (OR 3.11, CI 2.08-4.65) and postoperative pneumonia (OR 7.04, CI 1.95-25.45).

Conclusion

Variables identified above can be used to predict the cohort of EVAR patients which will likely require ICU for ≥1 day. Development of postoperative pneumonia is associated with a 7.04 times increase in ICU LOS ≥1 day.

## Introduction

The past two decades have seen a revolution in the surgical treatment of abdominal aortic aneurysms (AAA). Since EVAR was first described by Parodi et al., subsequent randomized controlled trials have supported its safety and efficacy [1-3]. EVAR has now become the most common modality for the treatment of abdominal aortic aneurysms. One of the main advantages of EVAR over traditional open AAA repair is the reduced intensive care unit (ICU) and hospital length of stay (LOS). There is recent evidence to suggest that EVAR can be performed safely even in the outpatient settings [4]. In the era of increasing focus on reducing the postoperative complications, the length of stay in the intensive care unit and hospital, and healthcare costs, hospitals are developing strategies to improve these metrics. 

Most patients do not need ICU care after elective EVAR. However, patients with numerous physiologic risk factors and those who undergo emergent EVAR are in need of ICU care postoperatively. Increased length of ICU stay after EVAR has been shown to be associated with significant complications, including death [5]. In this study, we aimed to determine the associating factors and develop a risk model for predicting the increased ICU LOS after EVAR. For this purpose, we chose to use the American College of Surgeons National Surgical Quality Improvement Program (ACS-NSQIP), since this is the largest surgical database in the US with a record of ICU LOS for a variety of general and vascular surgery operations.

## Materials and methods

Dataset

For the purpose of this study, we retrospectively analyzed the database provided by the American College of Surgeons National Surgical Quality Improvement Program (ACS-NSQIP). This database provides the users with Participant Use Files (ACS-NSQIP PUFs) [6]. These files contain patient de-identified data from multiple hospitals that participate in the ACS-NSQIP. ACS ensures that the information in the database remains compliant with the requirements of the Health Insurance Portability and Accountability Act (HIPAA). The database contains data from over 250 hospitals across the United States. It contains over 150 patient variables, including preoperative, intraoperative and postoperative factors. In order to ensure the integrity of the data, ACS requires each site to hire a reviewer who is specifically trained to collect and enter the data into the database. Data are collected in a systematic sampling process using an eight-day cycle schedule to avoid bias in selecting cases. To maintain diversity and high-quality data, high volume procedures are limited to a certain maximum number of cases per cycle, and cases with incomplete 30-day follow up are excluded from the database. The database is reliable with the inter-rater reliability (IRR) audit of the overall disagreement rate of approximately 2% among the participating sites [7]. Methods used for this analysis have been described in the previously published literature [7-11]. 

Outcomes

The main ACS NSQIP PUF was merged with the EVAR-Procedure Targeted PUF to identify patients who underwent EVAR in the calendar year 2015. Patients were divided into two groups based on LOS in the ICU: Group 1 (ICU LOS < 1 day) and Group 2 (ICU LOS ≥1 day). Several pre-operative, intra-operative and post-operative variables were compared between these two groups.

Statistical analysis

All variables were summarized prior to any analysis using frequencies and percentages or means, medians, and standard deviations to examine data quality. The distribution of continuous variables was checked using histograms, box plots, and normal probability plots. Logistic regression was applied using a bivariate approach to search for associations between ICU LOS ≥1 and potential predictor variables. Because there were so many significant variables, only variables with a significant association with ICU LOS ≥1 having a *p*-value <0.01 were chosen as a subset for consideration for a multivariable predictor model. Before model selection was initiated, a check for multicollinearity between the potential predictor variables was performed using a variance inflation factor (VIF) statistics. The remaining predictors were then pared down to a final model using several methods of selection: best subsets, stepwise, backward, and forward. Inclusion and exclusion criteria for the selection methods that used them were set at *p* < 0.01 to be more stringent, given the large number of significant predictors. The results of these selection methods were combined to choose the most significant set of predictors of ICU LOS ≥1 collectively. The fit of the final model was assessed using Pearson, Deviance, and the Hosmer and Lemeshow goodness-of-fit tests. Model-adjusted odds ratios were used to quantify the size and direction of the significant associations. Predicted probabilities based on patient characteristics were calculated using the prediction equation created based on the model parameter estimates. All analyses were carried out using SAS version 9.4 (SAS Institute, Cary, NC).

## Results

Demographics

A total of 2,512 patients underwent EVAR in 2015. The mean age was 73.8 ± 9.0 years. ICU LOS could be determined in only 2,468 (18.7% females, 81.3% males) of these patients and were included in the analysis. Of these 2,468 patients, 1535 had ICU LOS<1 day (Group 1) and 933 had ICU LOS ≥1 day (Group 2). The mean ICU LOS was 1.0 (±2.8) days. 

Bivariate analysis

The following factors were found to have significant association with ICU LOS ≥1 day: sex (female vs male: odd ratio (OR) 1.26, confidence interval (CI) 1.03-1.55, *p *= 0.028, ), race (Hispanic vs non-Hispanic white: OR 3.93, CI 1.95-7.92, *p *< 0.001), body mass index (BMI; ≥30 vs <25: OR 0.77, CI 0.63-0.95, *p *= 0.004), indication for surgery (ruptured vs non-ruptured: OR 9.86, CI 6.65-14.64, *p *< 0.001), transferred status (OR 4.57, CI 3.54-5.91, *p *< 0.001), emergency surgery (OR 4.71, CI 3.87-5.73, *p *< 0.001), dependent functional health status prior to surgery (OR 2.48, CI 1.56-3.94, *p *< 0.001), history of chronic obstructive pulmonary disease (COPD; OR 1.58, CI 1.29-1.93, *p *< 0.001), congestive heart failure (CHF; OR 3.58, CI 2.03-6.31, *p *< 0.001), hypertension (HTN) requiring medication (OR 1.27, CI 1.04-1.56, *p *= 0.022), preoperative transfusion (OR 8.41, CI 4.49-15.75, *p *< 0.001), systemic sepsis (OR 6.43, CI 3.89-10.62, *p *< 0.001), wound classification (clean vs not clean OR 0.24, CI 0.09-0.62, *p *= 0.003), surgeon’s specialty (non-vascular vs vascular: OR 2.00, CI 1.23-3.25, *p* = 0.005), principal operative procedure (EVAR with aorto-aortic tube prosthetic vs EVAR with modular bifurcated prosthetic with one docking limb: OR 2.19, CI 1.52-3.14, *p *< 0.001), American Society of Anesthesiologists (ASA) classification (class 4-5 vs class 1-2: OR 4.69, CI 2.73-8.06, *p *< 0.001), rupture of aneurysm (OR 14.91, CI 1.89-117.67, *p* = 0.010), aneurysm diameter (cm; >6.0 vs ≤5.0: OR 1.50, CI 1.19-1.88, *p *< 0.001), proximal aneurysm extent (others vs infrarenal: OR 2.73, CI 2.08-3.58, *p *< 0.001), distal extent (internal iliac vs aortic: OR 2.40, CI 1.74-3.30, *p *< 0.001), access vessels (OR 2.25, CI 1.51-3.35, *p *< 0.001), access (bilateral groin cutdown vs percutaneous bilateral: OR 1.48, CI 1.25-1.76, *p *< 0.001), main body device (regular EVARs vs Cook Zenith Fenestrated: OR 0.47, CI 0.29-0.75, *p *= 0.001), iliac branched device (OR 1.37, CI 1.09-1.72, *p *= 0.007), aortic stent (OR 1.89, CI 1.38-2.59, *p *< 0.001), renal stent (OR 3.35, CI 2.45-4.57, *p *< 0.001), hypogastric revascularization (OR 3.12, CI 2.22-4.39, *p *< 0.001), lower extremity revascularization (OR 2.00, CI 1.37-2.92, *p *< 0.001), ischemic colitis (OR 42.11, CI 5.71-310.39, *p *< 0.001), lower extremity ischemia (OR 2.13, CI 1.14-3.97, *p *= 0.017), operation time (minutes; ≥180 vs <90: OR 3.89, CI 3.03-5.00, *p *< 0.001), discharge destination (home vs expired: OR 8.33, CI 4.42-15.72, *p *< 0.001), deep incisional surgical site infection (SSI; OR 11.59, CI 1.42-94.32, *p *= 0.022), pneumonia (OR 17.14, CI 6.11-48.08, *p *< 0.001), pulmonary embolism (OR 11.59, CI 1.42-94.32, *p *= 0.022), acute renal failure (OR 38.69, CI 5.23-286.45, *p *< 0.001), cerebrovascular accident (CVA)/stroke (OR 7.47, CI 1.61-34.63, *p *= 0.010), cardiac arrest (OR 4.29, CI 2.12-8.65, *p *< 0.001), myocardial infarction (MI; OR 9.64, CI 4.03-23.04, *p *< 0.001), bleeding requiring transfusion (OR 9.16, CI 6.80-12.33, *p *< 0.001), deep vein thrombosis (DVT; OR 9.99, CI 2.23-44.71, *p *= 0.003), sepsis (OR 4.15, CI 1.30-13.26, *p *= 0.017), return to the operation room (OR 3.12, CI 2.15-4.52, *p *< 0.001), and unplanned readmission (OR 2.27, CI 1.68-3.07, *p *< 0.001; Table 1).

**Table 1:** Patient characteristics by ICU LOS ICU LOS, intensive care unit length of stay; OR, odds ratio; CI, confidence interval; BMI, body mass index; COPD, chronic obstructive pulmonary disease; EVAR, endovascular aneurysm repair; EVAR AORTO-UNILIAC/AORTO-UNIFEM PROSTH, EVAR with aorto-uniiliac/aorto-unifemoral prosthesis; EVAR W/AORTO-AORTIC TUBE PROSTH, EVAR with aorto-aortic tube prosthesis; EVAR W/MDLR BFRC PROSTHE 1 LIMB, EVAR with modular bifurcated prosthesis with one docking limb; EVAR W/MDLR BFRC PROSTH 2 LIMBS, EVAR with modular bifurcated prosthesis with two docking limbs; EVAR W/UNIBDY BFRC PROSTH, EVAR with unibody bifurcated prosthesis; PLMT XTN PROSTH EVAR/DSJ 1st VSL, placement of extension prosthesis for endovascular repair of infrarenal abdominal aortic or iliac aneurysm, initial vessel; ASA, American Society of Anesthesiologists; SSI, surgical site infection; CVA, cerebrovascular accident; CPR, cardiopulmonary resuscitation; DVT, deep vein thrombosis

Variable	Total (N = 2468)	Group 1 ICU LOS <1 day (N = 1535)	Group 2 ICU LOS ≥1 day (N = 933)	OR (95% CI)*	P-value*	
Preoperative factors:						
Age (years)						
<60	138 (5.6)	76 (55.1)	62 (44.9)	Reference		0.166
60-69	638 (25.9)	388 (60.8)	250 (39.2)	0.79 (0.55, 1.15)		
70-79	1001 (40.5)	642 (64.1)	359 (35.9)	0.69 (0.48, 0.98)		
≥80	691 (28)	429 (62.1)	262 (37.9)	0.75 (0.52, 1.08)		
Sex						
Female	461 (18.7)	266 (57.7)	195 (42.3)	1.26 (1.03, 1.55)		0.028
Male	2007 (81.3)	1269 (63.2)	738 (36.8)	Reference		
Race						
Hispanic	40 (2.0)	11 (27.5)	29 (72.5)	3.93 (1.95, 7.92)		<0.001
Non-Hispanic Black	133 (6.6)	64 (48.1)	69 (51.9)	1.61 (1.13, 2.29)		
Non-Hispanic Other	31 (1.5)	14 (45.2)	17 (54.8)	1.81 (0.89, 3.70)		
Non-Hispanic White	1821 (89.9)	1090 (59.9)	731 (40.1)	Reference		
BMI (kg/m^2)						
<25 (normal)	655 (27.5)	374 (57.1)	281 (42.9)	Reference		0.004
25-<30 (overweight)	930 (39.0)	605 (65.0)	325 (35.0)	0.72 (0.58, 0.88)		
>30 (obese)	798 (33.5)	505 (63.3)	293 (36.7)	0.77 (0.63, 0.95)		
Ruptured indication for surgery						
Ruptured	188 (7.7)	31 (16.5)	157 (83.5)	9.86 (6.65, 14.64)		<0.001
Non-ruptured	2255 (92.3)	1490 (66.1)	765 (33.9)	Reference		
Transferred						
Yes	311 (12.6)	95 (30.6)	216 (69.4)	4.57 (3.54, 5.91)		<0.001
No	2156 (87.4)	1440 (66.8)	716 (33.2)	Reference		
Elective surgery						
Yes	1874 (76.0)	1331 (71.0)	543 (29.0)	Reference		<0.001
No	593 (24.0)	203 (34.2)	390 (65.8)	4.71 (3.87, 5.73)		
Diabetes (with oral agents or insulin)						
No	2080 (84.3)	1308 (62.9)	772 (37.1)	Reference		0.081
Non-insulin dependent	304 (12.3)	184 (60.5)	120 (39.5)	1.11 (0.86, 1.41)		
Insulin dependent	84 (3.4)	43 (51.2)	41 (48.8)	1.62 (1.04, 2.50)		
Current smoker within one year						
Yes	827 (33.5)	495 (59.8)	332 (40.2)	1.16 (0.98, 1.38)		0.089
No	1641 (66.5)	1040 (63.4)	601 (36.6)	Reference		
Dyspnea						
Yes	378 (15.3)	222 (58.7)	156 (41.3)	1.19 (0.95, 1.48)		0.131
No	2090 (84.7)	1313 (62.8)	777 (37.2)	Reference		
Dependent functional health status prior to surgery						
Yes	76 (3.1)	31 (40.8)	45 (59.2)	2.48 (1.56, 3.94)		<0.001
No	2384 (96.9)	1503 (63.0)	881 (37.0)	Reference		
History of severe COPD						
Yes	471 (19.1)	251 (53.3)	220 (46.7)	1.58 (1.29, 1.93)		<0.001
No	1997 (80.9)	1284 (64.3)	713 (35.7)	Reference		
Congestive heart failure in 30 days prior to surgery						
Yes	56 (2.3)	18 (32.1)	38 (67.9)	3.58 (2.03, 6.31)		<0.001
No	2412 (97.7)	1517 (62.9)	895 (37.1)	Reference		
Hypertension requiring medication						
Yes	1959 (79.4)	1196 (61.0)	763 (39.0)	1.27 (1.04, 1.56)		0.022
No	509 (20.6)	339 (66.6)	170 (33.4)	Reference		
Currently on dialysis (pre-op)						
Yes	43 (1.7)	22 (51.2)	21 (48.8)	1.58 (0.87, 2.90)		0.136
No	2425 (98.3)	1513 (62.4)	912 (37.6)	Reference		
Steroid use for chronic condition						
Yes	94 (3.8)	54 (57.4)	40 (42.6)	1.23 (0.81, 1.87)		0.334
No	2374 (96.2)	1481 (62.4)	893 (37.6)	Reference		
Preoperative transfusion						
Yes	70 (2.8)	12 (17.1)	58 (82.9)	8.41 (4.49, 15.75)		<0.001
No	2398 (97.2)	1523 (63.5)	875 (36.5)	Reference		
Systemic sepsis						
Yes	93 (3.8)	20 (21.5)	73 (78.5)	6.43 (3.89, 10.62)		<0.001
No	2375 (96.2)	1515 (63.8)	860 (36.2)	Reference		
Wound classification						
Clean	2447 (99.1)	1529 (62.5)	918 (37.5)	0.24 (0.09, 0.62)		0.003
Not clean	21 (0.9)	6 (28.6)	15 (71.4)	Reference		
Intra-operative factors:						
Prior abdominal aortic surgery						
Yes	659 (29.7)	396 (60.1)	263 (39.9)	1.17 (0.97, 1.41)	0.102	
No	1562 (70.3)	996 (63.8)	566 (36.2)	Reference		
Surgeon’s specialty						
Vascular	2400 (97.2)	1504 (62.7)	896 (37.3)	Reference	0.005	
Non-vascular	68 (2.8)	31 (45.6)	37 (54.4)	2.00 (1.23, 3.25)		
Principal operative procedure						
EVAR AORTO-UNILIAC/AORTO-UNIFEM PROSTH	105 (4.3)	58 (55.2)	47 (44.8)	1.35 (0.90, 2.02)	<0.001	
EVAR W/AORTO-AORTIC TUBE PROSTH	134 (5.4)	58 (43.3)	76 (56.7)	2.19 (1.52, 3.14)		
EVAR W/MDLR BFRC PROSTH 1 LIMB	1091 (44.2)	682 (62.5)	409 (37.5)	Reference		
EVAR W/MDLR BFRC PROSTH 2 LIMBS	793 (32.1)	549 (69.2)	244 (30.8)	0.74 (0.61, 0.90)		
EVAR W/UNIBDY BFRC PROSTH	198 (8.0)	106 (53.5)	92 (46.5)	1.45 (1.07, 1.96)		
PLMT XTN PROSTH EVAR/DSJ 1^ST^ VSL	147 (6.0)	82 (55.8)	65 (44.2)	1.32 (0.93, 1.87)		
Principal anesthesia technique						
General	2204 (89.4)	1362 (61.8)	842 (38.2)	1.18 (0.90, 1.55)	0.225	
Non- general	262 (10.6)	172 (65.6)	90 (34.4)	Reference		
ASA classification						
1-2, no disturbance-mild disturbance	92 (3.7)	75 (81.5)	17 (18.5)	Reference	<0.001	
3, severe disturbance	1443 (58.7)	1008 (69.9)	435 (30.1)	1.90 (1.11, 3.26)		
4-5, life-threatening-moribund	924 (37.6)	448 (48.5)	476 (51.5)	4.69 (2.73, 8.06)		
Rupture of aneurysm						
Yes	10 (0.4)	1 (10.0)	9 (90.0)	14.91 (1.89, 117.67)	0.010	
No	2458 (99.6)	1534 (62.4)	924 (37.6)	Reference		
Aneurysm diameter (cm)						
≤5.0	576 (24.6)	356 (61.8)	220 (38.2)	Reference	<0.001	
5.0-5.5	667 (28.5)	479 (71.8)	188 (28.2)	0.64 (0.50, 0.81)		
5.5-6.0	455 (19.4)	309 (67.9)	146 (32.1)	0.77 (0.59, 0.99)		
>6.0	643 (27.5)	334 (51.9)	309 (48.1)	1.50 (1.19, 1.88)		
Proximal aneurysm extent						
Infrarenal	2074 (89.6)	1359 (65.5)	715 (34.5)	Reference	<0.001	
Others (Juxta-, para-, supra-renal, type IV thoracoabdominal aneurysm)	241 (10.4)	99 (41.1)	142 (58.9)	2.73 (2.08, 3.58)		
Distal extent						
Aortic	903 (47.3)	645 (71.4)	258 (28.6)	Reference	<0.001	
Common iliac	690 (36.2)	442 (64.1)	248 (35.9)	1.40 (1.13, 1.73)		
External iliac	129 (6.7)	69 (53.5)	60 (46.5)	2.17 (1.49, 3.16)		
Internal iliac	186 (9.8)	95 (51.1)	91 (48.9)	2.40 (1.74, 3.30)		
Access vessels (conduit, repair)						
Yes	102 (4.1)	44 (43.1)	58 (56.9)	2.25 (1.51, 3.35)	<0.001	
No	2366 (95.9)	1491 (63.0)	857 (37)	Reference		
Access						
Attempted percutaneous access converted to open cutdown	22 (0.9)	13 (59.1)	9 (40.9)	1.40 (0.59, 3.30)	<0.001	
Bilateral groin cutdown	1028 (42.0)	593 (57.7)	435 (42.3)	1.48 (1.25, 1.76)		
One groin cutdown	231 (9.4)	141 (61.0)	90 (39.0)	1.29 (0.96, 1.73)		
Percutaneous bilateral	1169 (47.7)	782 (66.9)	387 (33.1)	Reference		
Main body device						
Cook Zenith Fenestrated	74 (3.1)	33 (44.6)	41 (55.4)	Reference	0.001	
Regular EVARs	2350 (96.9)	1486 (63.2)	864 (36.8)	0.47 (0.29, 0.75)		
Iliac branched device						
Yes	357 (14.5)	199 (55.7)	158 (44.3)	1.37 (1.09, 1.72)	0.007	
No	2111 (85.5)	1336 (63.3)	775 (36.7)	Reference		
Aortic (bare metal) stent						
Yes	168 (6.8)	80 (47.6)	88 (52.4)	1.89 (1.38, 2.59)	<0.001	
No	2300 (93.2)	1455 (63.3)	845(36.7)	Reference		
Renal stent						
Yes	188 (7.6)	66 (35.1)	122 (64.9)	3.35 (2.45, 4.57)	<0.001	
No	2280 (92.4)	1469 (64.4)	811 (35.6)	Reference		
Hypogastric embolization						
Yes	175 (7.1)	100 (57.1)	75 (42.9)	1.25 (0.92, 1.71)	0.153	
No	2293 (92.9)	1435 (62.6)	858 (37.4)	Reference		
Hypogastric revascularization						
Yes	154 (6.2)	55 (36.2)	97 (63.8)	3.12 (2.22, 4.39)	<0.001	
No	2316 (93.8)	1480 (63.9)	836 (36.1)	Reference		
Lower extremity revascularization						
Yes	113 (4.6)	52 (46.0)	61 (54.0)	2.00 (1.37, 2.92)	<0.001	
No	2355 (95.4)	1483 (63.0)	872 (37.0)	Reference		
Ischemic colitis						
Yes	26 (1.1)	1 (3.9)	25 (96.1)	42.11 (5.71, 310.39)	<0.001	
No	2442 (98.9)	1534 (62.8)	908 (37.2)	Reference		
Lower extremity ischemia						
Yes	41 (1.7)	18 (43.9)	23 (56.1)	2.13 (1.14, 3.97)	0.017	
No	2427 (98.3)	1517 (62.5)	910 (37.5)	Reference		
Total operation time (minutes)						
<90	651 (26.4)	471 (72.3)	180 (27.7)	Reference	<0.001	
90-120	629 (25.5)	431 (68.5)	198 (31.5)	1.20 (0.95, 1.53)		
120-180	713 (28.8)	442 (62.0)	271 (38.0)	1.60 (1.28, 2.02)		
≥180	475 (19.3)	191 (40.2)	284 (59.8)	3.89 (3.03, 5.00)		
Postoperative factors:						
Discharge destination						
Expired	63 (2.6)	12 (19.1)	51 (80.9)	8.33 (4.42, 15.72)	<0.001	
Home	2173 (88.1)	1439 (66.2)	734 (33.8)	Reference		
Other	228 (9.3)	83 (36.4)	145 (63.6)	3.43 (2.58, 4.55)		
Open wound/wound infection						
Yes	31 (1.3)	14 (45.2)	17 (54.8)	2.02 (0.99, 4.11)	0.054	
No	2437 (98.7)	1521 (62.4)	916 (37.6)	Reference		
Superficial surgical site occurrence						
Superficial surgical incisional SSI	26 (1.1)	14 (53.8)	12 (46.2)	1.42 (0.65, 3.07)	0.380	
No Complication	2442 (98.9)	1521 (62.3)	921 (37.7)	Reference		
Deep incisional SSI						
Deep incisional SSI	8 (0.3)	1 (12.5)	7 (87.5)	11.59 (1.42, 94.32)	0.022	
No complication	2460 (99.7)	1534 (62.4)	926 (37.6)	Reference		
Wound disruption						
Wound disruption	3 (0.1)	1 (33.3)	2 (66.7)	3.30 (0.30, 36.38)	0.331	
No complication	2465 (99.9)	1534 (62.2)	931 (37.8)	Reference		
Pneumonia						
Pneumonia	44 (1.8)	4 (9.1)	40 (90.9)	17.14 (6.11, 48.08)	<0.001	
No complication	2424 (98.2)	1531 (63.2)	893 (36.8)	Reference		
Pulmonary embolism						
Pulmonary embolism	8 (0.3)	1 (12.5)	7 (87.5)	11.59 (1.42, 94.32)	0.022	
No complication	2460 (99.7)	1534 (62.4)	926 (37.6)	Reference		
Urinary tract infection						
Urinary tract infection	24 (1.0)	11 (45.8)	13 (54.2)	1.96 (0.87, 4.39)	0.103-	
No complication	2444 (99.0)	1524 (62.4)	920 (37.6)	Reference		
Acute renal failure						
Acute renal failure	24 (1.0)	1 (4.2)	23 (95.8)	38.69 (5.23, 286.45)	<0.001	
No complication	2444 (99.0)	1534 (62.8)	910 (37.2)	Reference		
CVA/stroke with neurological deficit						
CVA/stroke	11 (0.5)	2 (18.2)	9 (81.8)	7.47 (1.61, 34.63)	0.010	
No complication	2457 (99.5)	1533 (62.4)	924 (37.6)	Reference		
Cardiac arrest requiring CPR						
Cardiac arrest requiring CPR	39 (1.6)	11 (28.2)	28 (71.8)	4.29 (2.12, 8.65)	<0.001	
No complication	2429(98.4)	1524 (62.7)	905 (37.3)	Reference		
Myocardial infarction						
Myocardial infarction	40 (1.6)	6 (15.0)	34 (85.0)	9.64 (4.03, 23.04)	<0.001	
No complication	2428 (98.4)	1529 (63.0)	899 (37.0)	Reference		
Bleeding transfusion						
Transfusion intraoperative/postoperative	309 (12.5)	59 (19.1)	250 (80.9)	9.16 (6.80, 12.33)	<0.001	
No complication	2159 (87.5)	1476 (68.4)	683 (31.6)	Reference		
DVT/thrombophlebitis						
DVT requiring therapy	14 (0.6)	2 (14.3)	12 (85.7)	9.99 (2.23, 44.71)	0.003	
No complication	2454 (99.4)	1533 (62.5)	921 (37.5)	Reference		
Sepsis						
Sepsis	14 (0.6)	4 (28.6)	10 (71.4)	4.15 (1.30, 13.26)	0.017	
No complication	2454 (99.4)	1531 (62.4)	923 (37.6)	Reference		
Return to operation room						
Yes	128 (5.2)	46 (35.9)	82 (64.1)	3.12 (2.15, 4.52)	<0.001	
No	2340 (94.8)	1489 (63.6)	851 (36.4)	Reference		
Unplanned readmission						
Yes	188 (7.6)	82 (43.6)	106 (56.4)	2.27 (1.68, 3.07)	<0.001	
No	2280 (92.4)	1453 (63.7)	827 (36.3)	Reference		
* All odds ratios and p-values are from binomial logistic regression modeling ICU LOS ≥1, exact logistic regression used as needed. When an association is significant (p-value), odds ratios with 95% confidence limits not including 1 are considered significant.						

Multivariable analysis

The following factors were found to have significant association with ICU LOS ≥1 day: ruptured AAA (OR 3.88, CI: 1.97-7.65, *p *< 0.001), aneurysm ≤5 cm (vs. >6.0 cm: OR 1.50, CI: 1.12-2.02, *p *< 0.001), ASA score of 4-5 (vs. 1-2: OR 2.82, CI: 1.50-5.31, *p *< 0.001), operative time ≥180 minutes (vs <90 minutes: OR 2.10, CI: 1.51-2.93, *p *< 0.001), bilateral groin cut downs (vs. percutaneous bilateral: OR 1.37, CI: 1.10-1.71, *p *= 0.006), non-infrarenal AAA (OR 1.65, CI: 1.16-2.35, *p *= 0.005), renal artery stent (OR 2.13, CI: 1.42-3.21, *p *< 0.001), aortic stent (OR 2.39, CI: 1.60-3.55, *p *< 0.001), emergency surgery (OR 2.56, CI: 1.94-3.38, p<0.001), need for blood transfusion (OR 3.11, CI: 2.08-4.65, *p *< 0.001), postoperative pneumonia (OR 7.04, 1.95-25.45, *p *= 0.003), and unplanned readmission (OR 1.98, CI: 1.35-2.91, *p* < 0.001; Table 2). 

**Table 2:** Final multivariable model for ICU LOS ≥1 day ICU LOS, intensive care unit length of stay; OR, odds ratio; CI, confidence interval; AAA, abdominal aortic aneurysm; ASA, American Society of Anesthesiologists

Risk factor	OR (95% CI)*	P-value*
Elective surgery		
Yes	Reference	<0.001
No	2.56 (1.94, 3.38)	
Indication for Surgery		
Ruptured AAA	3.88 (1.97, 7.65)	<0.001
Non-ruptured AAA	Reference	
Operation time (minutes)		
<90	Reference	<0.001
90-120	1.14 (0.85, 1.52)	
120-180	1.27 (0.95, 1.70)	
≥180	2.10 (1.51, 2.93)	
Aneurysm diameter (cm)		
≤5.0	1.50 (1.12, 2.02)	<0.001
5.0-5.5	0.84 (0.62, 1.12)	
5.5-6.0	0.86 (0.63, 1.19)	
>6.0	Reference	
ASA classification		
1-2, no disturbance-mild disturbance	Reference	<0.001
3, severe disturbance	1.69 (0.90, 3.14)	
4-5, life-threatening-moribund	2.82 (1.50, 5.31)	
Access		
Attempted percutaneous access converted to open cut-down	0.44 (0.10, 1.90)	0.006
Bilateral groin cut-down	1.37 (1.10, 1.71)	
One groin cut-down	0.84 (0.57, 1.24)	
Percutaneous bilateral	Reference	
Proximal aneurysm extent		
Infrarenal	Reference	0.005
Others (Juxta-, para-, supra-renal, type IV thoracoabdominal aneurysm)	1.65 (1.12, 2.35)	
Renal stent		
Yes	2.13 (1.42, 3.21)	<0.001
No	Reference	
Aortic (bare metal) stent		
Yes	2.39 (1.60, 3.55)	<0.001
No	Reference	
Pneumonia		
Pneumonia	7.04 (1.95, 25.45)	0.003
No complication	Reference	
Bleeding transfusion		
Transfusions/ intraop/postop	3.11 (2.08, 4.65)	<0.001
No complication	Reference	
Unplanned readmission		
Yes	1.98 (1.35, 2.91)	<0.001
No	Reference	
* Multivariable logistic regression model for ICU LOS ≥1; odds ratios and p-values are adjusted for all other variables in the model/table.		

Predicted probability

The probability of ICU LOS ≥1 day was calculated for each factor identified from multivariable analysis. The probability of ICU LOS ≥1 day was 11.73% for patients who underwent bilateral groin cut down, 12.72% for patients with aneurysm diameter ≤5cm, 13.82% for patients who had non-infrarenal proximal aneurysm, 16.14% for patients who needed unplanned readmission, 16.95% for patients whose operative time was ≥180 minutes, 17.14% for patients who needed renal stent, 18.81% for patients who needed aortic stent, 19.90% for patients who underwent EVAR as non-elective surgery, 21.48% for patients who had an ASA classification of 4-5, 23.17% for patients who needed transfusion (intra/postoperative), 27.36% for patients who had ruptured aneurysm as indication for surgery, and 40.57% for patients who had postoperative pneumonia. Patients who had all of these factors have a 99.99% probability of having ICU LOS ≥ 1 day after EVAR. Patients without any of these factors had a probability of 8.84% at baseline (Table 3).

**Table 3:** Predicted probabilities of ICU LOS ≥1 from a multivariable model ASA, American Society of Anesthesiologists; ICU LOS, intensive care unit length of stay

Bilateral groin cut-downs	Aneurysm diameter ≤5.0cm	No infrarenal proximal aneurysm	Unplanned readmission	Operation time ≥180mins	Renal stent	Aortic stent	Non-elective surgery	ASA 4-5	Bleeding transfusion intra/postop	Ruptured indication for surgery	Pneumonia	Probability of ICU LOS ≥1 (%)
-	-	-	-	-	-	-	-	-	-	-	-	8.84%
+	-	-	-	-	-	-	-	-	-	-	-	11.73%
-	+	-	-	-	-	-	-	-	-	-	-	12.72%
-	-	+	-	-	-	-	-	-	-	-	-	13.82%
-	-	-	+	-	-	-	-	-	-	-	-	16.14%
-	-	-	-	+	-	-	-	-	-	-	-	16.95%
-	-	-	-	-	+	-	-	-	-	-	-	17.14%
-	-	-	-	-	-	+	-	-	-	-	-	18.81%
-	-	-	-	-	-	-	+	-	-	-	-	19.90%
-	-	-	-	-	-	-	-	+	-	-	-	21.48%
-	-	-	-	-	-	-	-	-	+	-	-	23.17%
-	-	-	-	-	-	-	-	-	-	+	-	27.36%
-	-	-	-	-	-	-	-	-	-	-	+	40.57%
+	+	+	+	+	+	+	+	+	+	+	+	99.99%

## Discussion

This analysis of a large, multi-institutional database of all EVARs performed across the US aims to determine the factors associated with increased length of ICU stay during the postoperative period. It identifies the following risk factors: ruptured AAA, emergent surgery, longer operative time, higher ASA score, bilateral groin cut-downs, extension of aneurysm into the supra-renal aortic segment, need for aortic or renal stents, postoperative pneumonia, bleeding and unplanned readmission to hospital. The predicted probability model shows that in the absence of these risk factors, the probability of prolonged ICU admission is 8.8%, and in the presence of all of these risk factors, the predicted probability of prolonged ICU stay is 99.99%. Presence of post-operative pneumonia itself is associated with a 41% probability of prolonged ICU admission. These findings have potentially important implications for the management of EVAR patients. 

Our study shows several important factors that contribute to prolonged ICU stay after EVAR (Table 2). While some of these factors can be considered modifiable, several of them cannot be modified. For example, variables such as ruptured AAA, high ASA scores and juxta-renal AAAs cannot be changed. On the other hand, factors such as bilateral femoral cut-downs, prolonged operative time and postoperative pneumonia can be considered potentially preventable. For factors that cannot be modified, this study highlights the importance of recognizing patients who are at high risk for requiring prolonged ICU stay. It also highlights risk factors that can be prevented to reduce ICU LOS. Postoperative pneumonia is associated with an extremely high risk for prolonged ICU stay after EVAR.

Endovascular aneurysm repair vs open repair (EVAR trial 1) and the Dutch randomized trial comparing conventional and endovascular repair of AAA (DREAM Trial) have established EVAR as a safe and effective alternative surgical treatment for AAA [2-3]. Among many advantages of EVAR over open repair are the reduced operative time, length of ICU stay, length of hospital stay, and early ambulation. In addition, EVAR is more cost-effective as compared to open AAA repair. While the cost of endografts is the major contributor toward the overall cost of EVAR, major reduction in the cost comes from reduced ICU LOS and hospital LOS. During the past decade, the overall cost associated with EVAR has improved, and most of this cost reduction comes from reduced LOS [12]. Our intent for this study was to examine all factors associated with ICU LOS and to focus on those factors, which are deemed preventable. Our study shows that the development of postoperative pneumonia is a significant preventable factor. Strategies to improve preventable factors may help us reduce the ICU LOS, associated morbidities, and overall cost of EVAR. Prolonged ICU LOS after EVAR itself has been associated with the development of serious complications, including death [5]. With an increasing focus on reducing postoperative complications and stress on using cost-effective strategies, our analysis identifies an area of improvement for patients undergoing EVAR. Postoperative complications impose a tremendous financial burden on both the patient and the healthcare system [13-14]. A retrospective review of the veteran affairs (VA) surgical patients showed that the estimated excess cost from postoperative complications ranged from $8,338 for superficial surgical site infection to $29,595 for failure to wean from ventilation within 48 hours. For pneumonia itself, the estimated excess cost was $12,798 [14]. Postoperative complications are highly associated with readmission, and readmission comes with additional cost. Lawson et al. show that by 5% reduction in postoperative complications, a potential 2,092 readmissions can be prevented, saving Medicare 31 million dollars per year [15].

Patients undergoing major vascular surgery operations are at significantly high risk for developing postoperative complications compared to those who undergo other major surgical operations [16]. Postoperative morbidity is associated with a significantly high risk of mortality [17]. Association of postoperative morbidity is, in fact, higher than that of baseline comorbid disease [18]. While the other complications after EVAR have been described in literature before, there is sparse literature on the impact of postoperative pneumonia on the overall prognosis. A retrospective review of all vascular surgery operations performed across the US has shown that postoperative pneumonia is associated with a significantly high risk of developing end-organ dysfunction, 30-day mortality, prolonged length of hospital stay and hospital readmission [19]. Our study shows that the development of postoperative pneumonia is associated with increased LOS in the ICU in the postoperative period. These findings are important, as postoperative pneumonia can be considered preventable for elective operations. Katsura et al. have shown that respiratory muscle training in the preoperative time period can reduce the incidence of postoperative pulmonary complications among patients undergoing cardiac and abdominal operations [20]. Guay et al. have demonstrated that intraoperative use of low volume ventilation reduces the incidence of postoperative respiratory complications [21]. Unfortunately, ACS-NSQIP data does not have information available regarding the ventilator settings during the operations. Popping et al. document the benefit of epidural analgesia on decreasing postoperative pulmonary morbidity [22]. Ireland et al. have stressed the importance of continuous airway pressure during the postoperative period to prevent postoperative morbidity [23]. Such strategies can be employed to prevent postoperative pneumonia and hence the complications associated with it. There is evidence to support that development of pneumonia prevention bundles by hospitals reduces the incidence of pneumonia [24-25]. The findings from our study, in combination with the above-mentioned literature, make the case for employing all pneumonia prevention strategies for EVAR patients to reduce the incidence of this complication and the associated increased length of ICU stay. As shown by Greenblatt et al., reducing the incidence of pneumonia after aortic surgery can also help reduce the incidence of unplanned readmission to the hospital [26].

This study has several limitations. It is a retrospective analysis where findings are limited to those hospitals that were enrolled in ACS-NSQIP in 2015. In addition, the data is not all-inclusive, and it is a self-reporting registry. We lack information about the documented reason for ICU admission. The strengths of this study are that it is based on the largest surgical database in the US. The findings from this database in previously published literature have been shown to be reliable and reproducible in subsequent studies. The large volume of patients in the dataset provides a powerful tool for meaningful statistical analysis. It includes large, tertiary care academic medical centers and small community hospitals, hence providing data from a broad variety of sources. Correlation of pneumonia with increased ICU LOS and other morbidities correlates with previously published literature. Our data shows that the occurrence of more than one complication is associated with longer length of ICU stay.

## Conclusions

With EVAR becoming the predominant modality for surgical treatment of AAA, attention should be paid to factors associated with increased morbidity and mortality after EVAR. Prolonged ICU LOS is associated with poor outcomes and increased cost. This study identifies the factors associated with prolonged ICU LOS after EVAR and identifies that postoperative pneumonia is associated with a significant risk of increased ICU LOS.
